# Theoretical and Experimental Investigation on the Nanostructures Evolution on Pre-Patterned Fused Silica by Focused Ion Beam

**DOI:** 10.3390/mi16111243

**Published:** 2025-10-31

**Authors:** Jianwei Ji, Yangsen Luo, Shaosen Liang, Jiyin Zhang, Kai Liu

**Affiliations:** 1 College of Mechanical Engineering, Chongqing University of Technology, Chongqing 400054, China; 2 School of Chemical Engineering & Technology, China University of Mining and Technology, Xuzhou 221116, China; 3 Department of Industrial Engineering, University of Padua, 35131 Padova, Italy

**Keywords:** self-organized nanostructures, ion sputtering, pre-patterned surface, fused silica

## Abstract

This paper investigates the laws governing the evolution of nanostructures on pre-patterned fused silica surfaces by energetic ion erosion. First, regular nanostructures are fabricated with the Focused Ion Beam (FIB) operating at optimized processing parameters. Then, as a function of the different ion fluences, the surface morphology evolution is studied on a surface with newly formed nanostructures. An experimental phenomenon of inter-transformation between nano-ripples and random dot-like structures is observed. In addition, the principles of the development of the nanostructures are analyzed theoretically. The simulation results fit well with the experiments. This work deeply studies the influence of the initial surface micro-morphology on the evolution of nanostructures, and is of great significance for the control of surface nanostructures generated by energetic ion sputtering.

## 1. Introduction

Highly regular nanostructured surfaces are attracting research consideration due to their unique optical, electrical, and magnetic properties. For example, a needle or cone-shaped array structure on a black silicon surface results in low reflection and strong absorption of visible and near-infrared light [1]. Such features significantly improve the sensitivity of photodetectors and the efficiency of solar cells. In addition, due to the deflection of light rays by surface nanostructures, Diffractive Optical Elements (DOEs) can produce an optical wavefront that conventional optical elements cannot realize [2]. Therefore, DOEs are widely used in holography, integrated optics, laser medicine, and so on. The surface nanostructures can also change the surface free energy and the contact angle to realize the wettability control [3] of the material surface and its potential applications, such as in micro-fluid devices, self-cleaning surfaces, and microelectronics. Additionally, surface nanostructures also have great application value in the fields of high-density storage [4], anti-reflective surfaces [5], and electrocatalysts [6].

In recent years, the fabrication and control of low-dimensional nanostructures on solid surfaces have received increasing attention and become a frontier research topic. In this regard, ion sputtering of a solid surface is one of the methods to generate self-organized nanostructures on a surface. A key advantage of ion irradiation is the spontaneous formation of nanostructures without a mask. Furthermore, the formation of ion irradiation-induced nanostructures has also been reported on various surfaces, such as films [7], metals [8], and semiconductors [9]. Therefore, it is of great significance to investigate the mechanism of ion irradiation-induced nanostructures formation and the evolution of the efficient and low-cost fabrication of surface nanostructures.

Since the phenomenon of surface nanostructures induced by ion sputtering was reported by Navez et al. in 1962, researchers have put tremendous effort into understanding the evolutionary mechanisms of resulting morphologies [10]. Previous studies have shown that the evolution of surface nanostructures is greatly influenced by the processing parameters of the ion beam, such as the fluence, incident energy, and ion incidence angle. However, most of the former research investigated the influence of ion beam processing parameters on surface morphology evolution of already smooth surfaces. Recently, Karmakar et al. [11] studied the relationship between the initial surface roughness and ion-induced surface morphology evolution on a Si surface. They prepared surfaces with varying initial roughness using chemical methods and by oblique angle bombarding of 16.7 keV O_2_^+^ ions. The difference in nanostructures and laws governing the evolution of surfaces with different roughness were analyzed emphatically. A similar study was conducted on Al film by Mishra et al. [12]. In addition, Toma et al. [13] bombarded a defocused Ar^+^ ion beam on a dielectric substrate, investigating the self-organized patterning evolution of polycrystalline noble metal films. They studied the laws governing the evolution of nanostructures on surfaces with different initial roughness with varying film thicknesses and performed a validation experiment with a polycrystalline Au and Ag film. According to the literature discussed, the ripples formed by incident beams highly depend on the preliminary surface topography. However, initial surfaces in the above experiments were uniform and randomly generated by ion sputtering or coating.

In contrast, a regular and pre-patterned surface is taken as the initial surface in this study. While various techniques such as photolithography can generate surface patterns, the focused ion beam (FIB) was selected for this fundamental study due to its unique advantages. Utilizing FIB for both the creation of the initial pattern and the subsequent irradiation ensures a perfectly self-contained process. This approach eliminates confounding variables inherent in combining different fabrication techniques (such as differing chemical contamination, thermal budgets, or damage mechanisms), thereby allowing us to isolate and scrutinize the effect of ion fluence on the evolution of a pre-defined topography with high purity. In addition, the law of ion beam sputtering and parameters influencing surface patterning are explored. Völlner et al. [14] also investigated the evolution of surface nanostructures on pre-patterned surfaces. They used a broad beam ion source with a low voltage (2000 eV). It is well known that the difference in voltage and divergence angle significantly influence the evolution of the surface morphologies. Therefore, in this paper, the experimental results of high voltage (30 keV) and focused ion beams are further discussed. In this paper, the nanostructure evolution mechanism is studied on a pre-patterned fused silica surface. The reason for choosing fused silica as experimental material is that it is a unique optical material with exclusive physical and chemical properties and is nearly irreplaceable in lens manufacturing and light delivery applications. Atomic force microscopy (AFM) studies the surface morphology, and the results are characterized and analyzed by the root mean square (RMS) of the surface roughness and calculating the power spectral density (PSD) function. The detailed experimental process is as follows. At first, laws governing the evolution of a fused silica surface processed by a FIB for different processing parameters are investigated. By employing a group of proper processing parameters, surfaces with regular nano-ripple structures are obtained. Then, the sample is rotated clockwise along the normal to the surface by 90° and irradiated again with FIB operated under the same parameters. The evolution of the surface morphology for different processing durations is analyzed. An experimental phenomenon of inter-transformation between the nano-corrugated structures and random dot-like structures is observed with AFM. In addition, the mechanisms governing the evolution of the nanostructures are analyzed theoretically, and the whole evolution process is completely described and verified by simulations.

## 2. Materials and Methods

The diameter of the fused silica substrates (Chengdu Qianghai Photoelectric Technology Co., Ltd., Chengdu, China) used in experiments is 8 mm. Substrates having very low surface roughness (<1 nm) with no apparent surface defects, as observed with the optical microscope, are used in experiments. Samples are gold coated to ensure good electrical conductivity of the surface.

**Figure 1:**
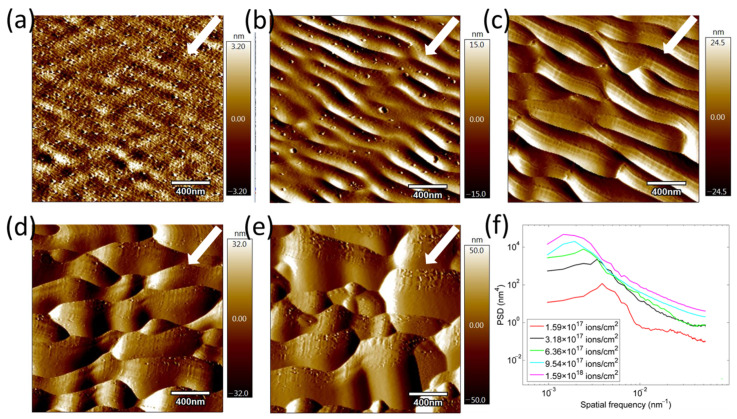
Morphologies and PSD of fused silica surfaces processed with FIB using different ion incident fluence (ions/cm^2^) (**a**) 1.59 × 10^17^; (**b**) 3.18 × 10^17^; (**c**) 6.36 × 10^17^; (**d**) 9.54 × 10^17^; (**e**) 1.59 × 10^18^; (**f**) PSD curves after different processing times. The area measured is 2 × 2 μm^2^, and the arrows indicate the direction of the incoming ions.

First, pre-patterned fused silica surfaces were produced. A series of experiments were conducted to obtain a group of processing parameters that can obtain very regular ripples. The experiments use an SEM-FIB dual-beam system (AURIGA from Zeiss, Oberkochen, Germany). Surface patterns over a predefined area of 0.1 × 0.2 mm^2^ are generated by rastering a Ga^+^ ion beam with characteristic 30 KeV incident energy, 750 nm beam spot, 16 nA current, and 3.622 A/cm^2^ current density. Jany et al. demonstrated that the growth dynamics of microstructures on the substrate are independent of the Ga^+^ implantation [15]. The FIB processes the samples for different ion fluences (1.59 × 10^17^ ions/cm^2^, 3.18 × 10^17^ ions/cm^2^, 6.36 × 10^17^ ions/cm^2^, 9.54 × 10^17^ ions/cm^2^, and 1.59 × 10^18^ ions/cm^2^) at an incident angle of 54°, with respect to the surface normal (sample surface is perpendicular to the electron beam gun at 54°). This angle was selected as it corresponds to the intrinsic geometry of our FIB-SEM system and, critically, lies within the range (typically 50–60°) predicted by the Bradley-Harper theory to be optimal for the formation of well-defined nanoripple patterns on amorphous surfaces like fused silica. The experimental results are shown in Figure 1. Initially, ripple wavelengths were very low, which increased gradually with ion fluence, ultimately increasing the peak-to-valley (P-V) values. For example, increasing ion incidence fluence from 1.59-to-6.36 × 10^17^ ions/cm^2^ decreases the regularity and causes bifurcations and truncations, as seen in Figure 1a–c. However, further increasing ion incidence fluence by an order from the initial value saturated the ripples, as seen in Figure 1d,e. These results are aligned well with those reported by Datta [16] and Chini [17]. Therefore, an irradiation fluence of 6.36 × 10^17^ ions/cm^2^ is chosen to produce the pre-patterned surface.

**Figure 2:**
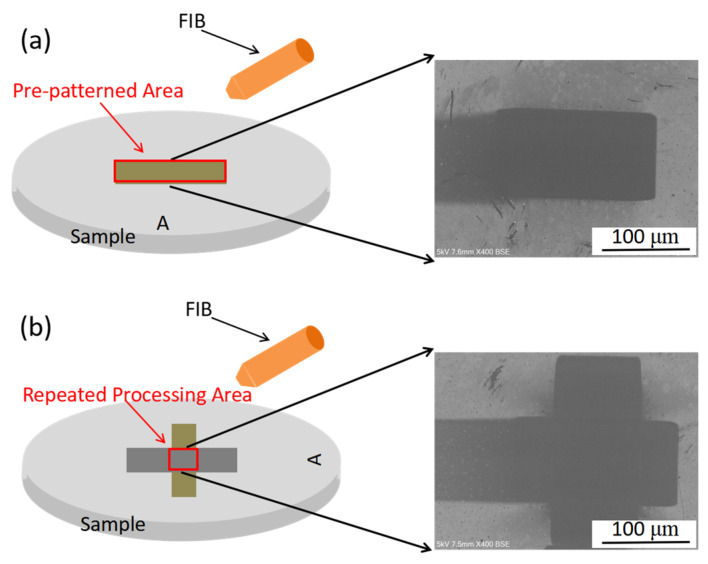
Schematic of experimental procedures: (**a**) pre-patterned surface area preparation; (**b**) the experiment producing the repeated processing area.

For the second irradiation, the samples are rotated clockwise along the normal to the surface by 90° and irradiated again with FIB operating under the same processing parameters (30 KeV, 16 nA, and 54°). Therefore, a repeated processing area develops between the second irradiation area and the area with pre-patterned nanostructures. The process of sample rotation and repeated processing area development is shown in Figure 2. Four pre-patterned areas are prepared with the same processing parameters. The first pre-patterned area is used as a control group. The second irradiation fluence of the other three pre-patterned areas is 3.18 × 10^17^ ions/cm^2^, 6.36 × 10^17^ ions/cm^2^, and 1.272 × 10^18^ ions/cm^2^. Then the morphology of the repeated processing area is measured and analyzed. Experiments are conducted at room temperature, and a clean environment is maintained to avoid contamination from the surroundings. Moreover, it is to be mentioned that after obtaining a pre-patterned surface, the role of the deposited gold plating layer can be neglected in the study of the evolution of a pre-patterned surface since the material removal thickness is far deeper than the gold plating thickness (<10 nm due to TEM micrograph).

The surface processed with FIB is further observed using AFM to seek its morphology. The AFM is operated under tapping mode to observe surface morphology under ambient conditions. The reconstruction algorithm eliminated the tip-sample dilation effect from the experimental results [18]. In addition, the PSD function calculated from the Fourier transform of the discrete data sets analyzed the surface roughness over the entire frame of spatial frequencies. The P-V and RMS are traditional surface evaluation parameters providing limited information on surface topography. A single parameter cannot entirely define the actual surface morphology, and the frequency information of the surface cannot be obtained comprehensively and effectively. Therefore, the PSD function is a predominately used optical surface evaluation parameter, providing in-depth and comprehensive information on surface morphology evolution [19,20,21,22].

## 3. Results

**Figure 3:**
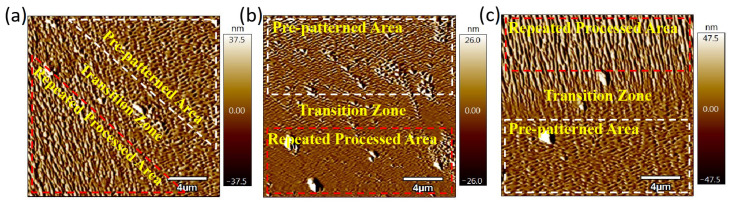
The repeated processing areas of the samples after FIB ion incidence fluence (ions/cm^2^): (**a**) 3.18 × 10^17^; (**b**) 6.36 × 10^17^; (**c**) 1.272 × 10^18^. The area measured is 20 × 20 μm^2^.

The area after processing is measured by AFM with a measurement range of 20 μm. The repeated processing areas (RPAs) and non-repeated processing areas are shown in Figure 3, where the processing ion fluence in Figure 3a–c are 3.18 × 10^17^ ions/cm^2^, 6.36 × 10^17^ ions/cm^2^, and 1.272 × 10^18^ ions/cm^2^, respectively. Figure 3a presents the surface that consists of the second radiation area and the repeated processing area, while Figure 3b,c present the surface consisting of the pre-patterned area and the repeated processing area. As seen from Figure 3, the width of the transition zone in two different areas is approximately several microns, which are related to the size of the FIB spot. Solid surface nanostructures can greatly affect the surface physicochemical properties such as the contact angle and absorptance/transmittance [23,24,25,26]. Therefore, the FIB sputtering method can be used to control the local physicochemical properties of the fused silica surface.

**Figure 4:**
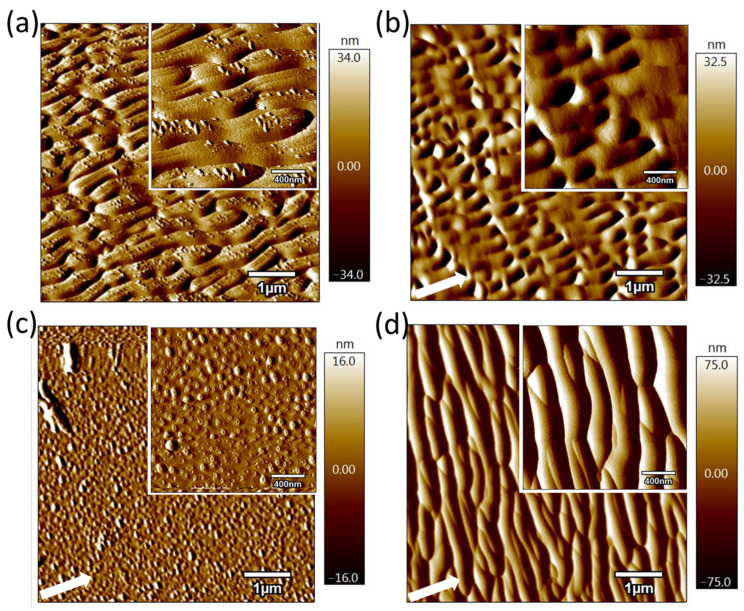
Morphology of the fused silica surface sputtered by FIB: (**a**) pre-patterned surface processed with ion fluence of 6.36 × 10^17^ ions/cm^2^; (**b**) 3.18 × 10^17^ ions/cm^2^; (**c**) 6.36 × 10^17^ ions/cm^2^; (**d**) 1.272 × 10^18^ ions/cm^2^ after the samples are rotated clockwise along the normal to the surface by 90° (6 × 6 μm^2^). The area measured in inserts is 2 × 2 μm^2^. The arrows indicate the direction of the incoming ions.

Figure 4 provides a more detailed presentation of the post-irradiated surfaces. The image is measured over an area of 6 × 6 μm^2^ with 256 × 256 pixels. One pixel in the image equals 24 × 24 nm^2^ area on the sample. Figure 4a shows the pre-patterned surface produced by FIB with optimized processing parameters, while Figure 4b–d show the surface morphologies formed after the second irradiation subsequent to the sample rotation by 90°, where the irradiation fluences are 3.18 × 10^17^ ions/cm^2^, 6.36 × 10^17^ ions/cm^2^, and 1.272 × 10^18^ ions/cm^2^, respectively. The RMS values of the surface roughness of each area in Figure 4 are 17.12 nm, 16.25 nm, 7.92 nm, and 36.34 nm, respectively. Combining Figure 3 and Figure 4, periodic ripples on the pre-patterned surface gradually disappeared by further increasing the ion fluence.

When the second irradiation fluence is close to the processing fluence of the pre-patterned surface produced, random dot-like structures appear on the surface. The P-V value of the surface with random dot-like structures is much smaller than that of the initial corrugated surface, representing relatively a smooth surface. After further processing, the corrugated structure reappears on the surface, and a 90° rotation occurs between the reappearing corrugated structure and the pre-processed corrugated structure. It is difficult to obtain a smooth surface when an ion beam processes the fused silica surface with an oblique angle. However, these experimental results show that proper processing parameters can remove nano-ripple structures and make the surface relatively smooth again. The results are of great significance to the controllable adjustment of surface nanostructures and obtained smooth surfaces by oblique ion beam processing.

**Figure 5:**
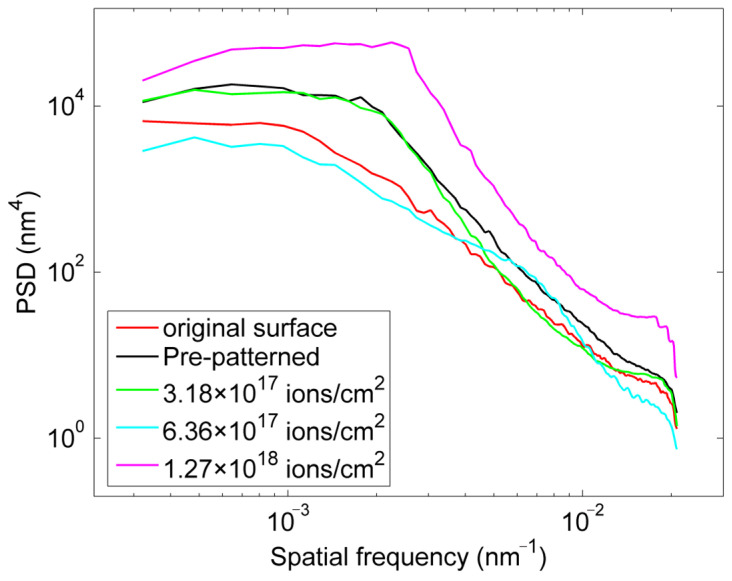
PSD curves of the original surface, the pre-patterned surface and surfaces processed with different ion fluence.

A quantitative analysis of the AFM data provides detailed geometric dimensions for the nanostructures. The pre-patterned ripples in Figure 4a exhibit an average spatial wavelength of ~550 nm, as determined from the dominant peak in the PSD function (Figure 5), and a peak-to-valley height of 25–55 nm from cross-sectional profile analysis. For the random dot-like structures in Figure 4c, statistical analysis using the AFM software’s particle analysis toolkit yields an average lateral diameter of 80–120 nm and a more precise average height of 8–15 nm. The size distribution of these nanodots is relatively uniform, with approximately 70% of features falling within ±20% of the mean dimensional values.

The PSD curves for different processing ion incidence fluence are shown in Figure 5. For comparison, the PSD curve for the unpatterned surface (after spray-gold) is also presented. Multiple measurements were taken to eliminate noise and error, and the mean results were obtained. The results show that the PSD curve decreases mainly in the higher spatial frequency band for a lower processing fluence. However, further increasing the processing ion fluence, the roughness of the higher spatial frequency band no longer changes, whereas a significant decline in the lower spatial frequency bands is observed. After a critical transition ion fluence, a new periodic structure perpendicular to the original corrugated structure appears. Notably, the most significant cause of the rapid deterioration of surface roughness and increase in the PSD curves is the appearance of the corrugated structure.

## 4. Discussion

Many theoretical studies have been carried out on self-organized nano-patterns on a solid surface induced by the irradiation of energetic ions [27,28,29,30]. Bradley and Harper (B–H) constructed a continuum theory based on Sigmund’s theory, successfully predicting the evolution of correlated surface features when amorphous materials are processed using ion irradiation. The B–H model explained the nano-pattern evolution process on the surface. These patterns emerge based on the competition of roughening with smoothing mechanisms induced by incident ions. Generally, the key surface roughening mechanisms is the Local Curvature-dependent Sputtering (LCS) mechanisms [31]. Thermally activated Surface self-Diffusion (TSD) [32], Ion enhanced viscous flow (IVF) [33], and ballistic smoothing [34] are the most widely used surface smoothing mechanisms. The B–H model can be represented by the following expression:

(1) $$\frac{\partial h}{\partial t}=-v_{0}+\kappa \frac{\partial h}{\partial x}+v_{x}\frac{\partial^{2}h}{\partial x^{2}}+v_{y}\frac{\partial^{2}h}{\partial y^{2}}-B\nabla^{4}h$$

where *h*(x,y,t) is the surface height at position (x,y) and time *t*, *ν*_0_ corresponds to erosion velocity of planar target, $\kappa \frac{\partial h}{\partial x}$ accounts for the dependence of sputtering yield on local surface slope along the ion beam direction, The terms $v_{x}\frac{\partial^{2}h}{\partial x^{2}}$ and $v_{y}\frac{\partial^{2}h}{\partial y^{2}}$ are the curvature-dependent roughening terms along the X and Y directions, respectively, and $-B\nabla^{4}h$ describes the surface smoothing mechanisms, such as thermally activated surface diffusion or ion-induced viscous flow, which tend to suppress high-frequency roughness. Since *ν*_0_ and *κ* remain constant and do not affect the surface structure characteristics during processing, they can be omitted from the equation. The direction and growth rate of periodic nanostructures is closely related to the values of *v*_x_ and *v*_y_.

In experiments producing pre-patterned surfaces, *v*_x_ is always larger than *v*_y_, which shows that the material removal is more dependent on the erosion rate along the X direction of the surface curvature than those along the Y direction, thus forming a periodic corrugated structure. In the second irradiation experiment on the pre-patterned surface, the sample is rotated by 90°. Consequently, the erosion rate dependence on the curvature of the surface in the Y direction affects the material removal rate more than those along the X direction. However, the total effect of the curvature-dependent erosion rates along the X and Y directions on the material removal remains the same with increasing ion fluence. When the secondary processing ion fluence is nearly similar to the primary processing ion fluence of the pre-patterned surface, the erosion rate becomes independent of the X and Y-directed curvatures, removing a similar amount of material in both directions. Therefore, random dot-like structures on the surface can be observed rather than periodic nanostructures in a certain direction.

The observed inter-transformation between ripple and dot-like structures, governed by the competition between anisotropic curvature-dependent sputtering (*ν*ₓ, *ν*_y_) and isotropic smoothing (*B*), is not unique to fused silica. Similar morphological transitions have been reported in other material systems. For instance, Ziberi et al. [35] observed that normal incidence irradiation of Si and Ge surfaces with a rotated broad Ar^+^ ion beam could suppress ripple formation and lead to isotropic dot patterns, a result analogous to our findings when the effective anisotropy is minimized. Similarly, Toma et al. [13] studied pattern evolution on polycrystalline Au and Ag films with varying initial roughness and found that the resulting morphology (ripples, dots, or a transitional state) was highly sensitive to the initial surface conditions and ion fluence, underscoring the generality of the competition between roughening and smoothing. These comparisons suggest that the underlying physical mechanism described by the B–H framework has a degree of universality across different amorphous and polycrystalline materials.

From a technological perspective, the ability to controllably induce transitions between ordered and disordered nanostructures, or even to achieve a relatively smooth surface from a pre-patterned one using ion beams, opens up significant possibilities. It could enable the in situ repair or smoothing of optical components (like lenses or phase plates made of fused silica) that have developed unwanted nano-ripples during fabrication or service. Furthermore, this control allows for the creation of surfaces with tunable and localized physicochemical properties. For example, one could design surfaces with specific regions exhibiting ripple-induced anisotropy (e.g., for directing cell growth or fluid flow) adjacent to regions with isotropic dot patterns or smoother areas, potentially useful in lab-on-a-chip devices, sensors, or templates for complex nanostructure growth.

Based on the preceding analysis, the classical B–H theory is used in simulations. The parameters in the B–H equation (*v*_x_, *v*_y_, and *B*) were selected through a combination of theoretical guidance and iterative calibration to achieve qualitative agreement with the experimental results, following established practices in the field. The Euler difference method with Δ*t* = 0.005 time step and Δ*x* = 0.5 spatial step is used in numerical simulations. Following footprints in Refs. [36,37], we used arbitrary units in our numerical calculations because many parameters in these equations involve unknown constants (e.g., diffusion and viscosity coefficients); however, the relative magnitudes and signs of the parameters are physically significant and control the pattern evolution.

**Figure 6:**
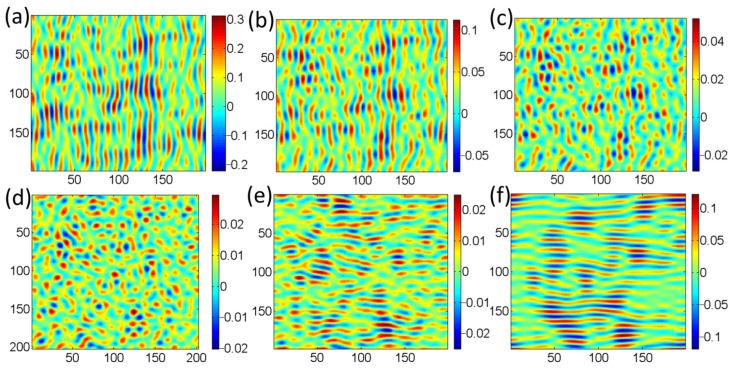
The evolution process of the surface nanostructures with the number of iterations: (**a**) pre-patterned surface at 1500; (**b**) 500; (**c**) 1000; (**d**) 1500; (**e**) 2500; and (**f**) 4500 after the samples are rotated clockwise along the normal to the surface by 90°.

The parameters for the simulation (*v*_x_ = 0.7, *v*_y_ = 0.15, *B* = 0.3) were selected and validated through a systematic process. Initially, the erosion parameters *v*_x_ and *v*_y_ were chosen based on the B–H theory’s requirement for a strong anisotropy (*v*_x_ > *v*_y_) to drive ripple formation at an oblique angle. The smoothing coefficient *B* was then iteratively calibrated to balance this roughening effect and prevent unrealistic surface divergence. The final parameter set was validated by its successful qualitative reproduction of the entire experimental morphological sequence: the transition from pre-patterned ripples to random dots and finally to the reappearance of ripples oriented 90° to the original. The consistency between the simulated and experimental evolution, particularly the similar ‘processing time’ needed for structure removal and reformation, confirms the appropriateness of the chosen parameters. The simulation results are given in Figure 6. Figure 6a shows the pre-patterned surface generated by 1500 iterations from an initially random surface, which is used as the initial surface for simulation. The RMS value is 0.0812. Then, the sample is rotated by 90° and processed for different times with the same processing parameters (the values of *v*_x_, *v*_y_, and *B* are constant in the simulation). The results shown in Figure 6b–f are obtained after 500 iterations, 1000 iterations, 1500 iterations, 2500 iterations and 4500 iterations, respectively, and the corresponding RMS values are 0.0258, 0.0111, 0.0068, 0.0073, and 0.0374, respectively.

Furthermore, the simulation results predicted that random dot-like structures are generated and the surface morphology gradually becomes less irregular compared to the initial periodic ripples with increasing iterations. When increasing iterations, the ripple structures reappear; however, reappearing ripple structures are perpendicular to the initial ripple structures. Moreover, the number of iterations required for the periodic structure to be completely removed is the same as the number of iterations needed to produce the pre-patterned structures. Similar conclusions are drawn from experimental data. Therefore, simulation results align well with the experimental data and validate the process.

As discussed, ion beam processing creates regular periodic ripples because the erosion rate is highly reliant on the curvature of the surface along the X direction and provides a higher material removal rate than those along the Y direction. In the previous section, we rotated the sample by 90°, used the same processing ion fluence, and observed the same material removal rate in both X and Y directions since the erosion rate becomes independent of the curvature direction. The control of the surface ripple structure in ion beam processing can be extended to uniform rotational processing with a continuous variation in *v*_x_ and *v*_y_. This processing method can overcome the direction singularity, and in theory, the formation of periodic corrugated structures can be avoided.

**Figure 7:**
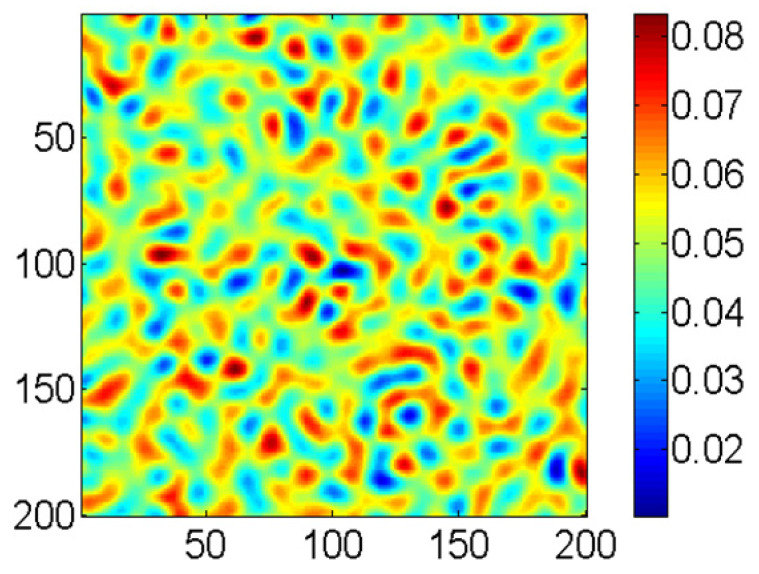
The simulated result of uniform rotational processing with 1500 iterations.

Based on the above analysis, uniform rotational processing is simulated. The simulation parameters used are the same as those used in the previous section: *v*_x_ = 0.7, *v*_y_ = 0.15, and *B* = 0.3. During the simulation, the rotation speed is defined as 1° per iteration. When the sample is rotated by 1°, the matrix of the surface is rotated by 1°. As shown in Figure 7, the number of iterations of the graph obtained by the simulation is 1500. The simulation results show that no periodic nanostructures are produced. The obtained simulation results align well with those reported by Ziberi et al. using a broad ion beam on Si and Ge surfaces [35].

## 5. Conclusions

Ion sputtering on a solid surface is one of the methods that is used to generate self-organized nanostructures on a surface. It is significant to study the evolution mechanism and achieve adjustable control of surface nanostructures induced by ions for the efficient and low-cost fabrication of surface nanostructures. It is well known that the periodic nano-corrugated structure will be produced on the smooth surface when energetic ions bombard the smooth surface with appropriate process parameters. However, the effect of the original micro-morphology on the subsequent structure development process has not been fully revealed. In this work, the laws governing the evolution of nanostructures by energetic ion erosion of pre-patterned surfaces have been investigated on fused silica. An experimental phenomenon of an inter-transformation between nano-corrugated structures and random dot-like structures is observed in the experiment. In addition, the mechanisms governing the evolution of the nanostructures are analyzed theoretically, and the whole evolution process is completely described by simulation. The simulation results align well with the experimental data and those reported in the literature. The findings suggest that the mechanism based on the competition between effective surface tensions (anisotropic roughening vs. isotropic smoothing) is potentially applicable to other material systems, such as semiconductors and metals, for achieving sophisticated morphological control. This study provides insights into the evolution of nanostructures when a pre-patterned surface is processed with an energetic ion beam. The demonstrated capability to reverse ripple formation and create smooth or isotropic nanostructures holds promise for applications requiring precise surface topography control, such as in situ smoothing of optical elements, fabrication of surfaces with graded functional properties, and developing templates for advanced photonic and sensing devices. Although this work utilized FIB for a controlled investigation, the theoretical framework suggests that the described evolution mechanism should be applicable to surfaces patterned by other means, such as photolithography or nanoimprinting. Therefore, a valuable future direction would be to verify the universality of these findings by employing a wider range of pre-patterning techniques and substrate materials (e.g., polymers or other glasses). In the long run, these results can help further research on the evolution and control of surface nanostructures generated by energetic ion sputtering.

## Data Availability

The original contributions presented in this study are included in the article. Further inquiries can be directed to the corresponding author.
